# Die WHO-Klassifikation von Hörverlusten: Ein Konsens zu einer deutschen Fassung

**DOI:** 10.1007/s00106-024-01494-z

**Published:** 2024-06-13

**Authors:** I. Holube, O. Dziemba, T. Fedtke, S. Hoth, O. Michel, K. Neumann, T. Rahne, D. Veraguth, P. von Gablenz, T. Wesarg, I. Baljić

**Affiliations:** 1 Institut für Hörtechnik und Audiologie, Jade Hochschule, Ofener Str. 16/19, 26121 Oldenburg, Deutschland; 2 Klinik und Poliklinik für Hals‑, Nasen‑, Ohrenkrankheiten, Kopf- und Halschirurgie, Universitätsmedizin Greifswald, Greifswald, Deutschland; 3 Physikalisch-Technische Bundesanstalt, Braunschweig, Deutschland; 4 Furtwänglerstr. 37, 69121 Heidelberg, Deutschland; 5 Institut für HNO-Begutachtung, St. Franziskus Hospital, 50825 Köln, Deutschland; 6 Klinik für Phoniatrie und Pädaudiologie, Universitätsklinikum Münster, Münster, Deutschland; 7 Klinik und Poliklinik für Hals‑, Nasen‑, Ohrenheilkunde, Kopf- und Halschirurgie, Universitätsmedizin Halle, Halle (Saale), Deutschland; 8 Klinik für Ohren‑, Nasen‑, Hals- und Gesichtschirurgie, Universitätsspital Zürich, Zürich, Schweiz; 9 Klinik für Hals‑, Nasen- und Ohrenheilkunde und Poliklinik, Universitätsklinikum Freiburg, Medizinische Fakultät, Albert-Ludwigs-Universität Freiburg, Freiburg, Deutschland; 10 Abteilung für Audiologie/Neurootologie, Klinik für Hals‑, Nasen- und Ohrenheilkunde, Plastische Operationen, Helios-Klinikum Erfurt, Erfurt, Deutschland

Im Jahr 2021 wurde im Gefolge einer neuen Resolution der Weltgesundheitsorganisation (WHO) zu Ohr- und Hörgesundheit der erste World Report on Hearing (WRH) publiziert [1]. Er soll die Integration von Maßnahmen zur Prävention, Diagnose und Behandlung von Hörstörungen und damit zusammenhängenden Ohrerkrankungen, welche mit einem Hörverlust einhergehen, in nationale Gesundheitspläne unterstützen. Der WRH fasst epidemiologische und ökonomische Daten zu Hörverlusten zusammen und umreißt verfügbare kosteneffiziente Lösungen. Das Ziel einer integrierten, patientenzentrierten Versorgung soll durch eine Reihe von Maßnahmen erreicht werden. Obwohl der WRH globale Reichweite hat, liegt ein besonderer Schwerpunkt auf Ländern mit niedrigen und mittleren Einkommen, in denen Menschen mit Hörverlust in der Regel unterversorgt sind. Der WRH wurde in Zusammenarbeit namhafter Experten erstellt; aus Deutschland hat Katrin Neumann, Münster, mitgearbeitet. Inzwischen wurde der WRH in Spanisch, Italienisch und Chinesisch übersetzt. Eine deutsche Übersetzung liegt auf der Webseite der WHO bislang nicht vor. Die Autorinnen und Autoren dieses Beitrags erarbeiteten einen Konsens zur Übertragung eines Kernbestandteils des WRH, der Klassifikation von Hörverlusten, ins Deutsche.

## Motivation für eine deutsche Fassung

Da in Deutschland die englische Sprache im akademischen Bereich weithin genutzt wird und auch breiten Bevölkerungsschichten geläufig ist, erscheint auf den ersten Blick eine Übertragung ins Deutsche nicht notwendig. Jedoch werden in Ausbildung, Studium und deutschsprachigen Schriften, wie z. B. in dieser Zeitschrift, und auch bei der verbalen Kommunikation in Praxis und Wissenschaft hierzulande deutschsprachige Begriffe verwendet, die eindeutig definiert sein sollten. Zudem weist der aktuelle WRH zusätzliche Schwerhörigkeitsabstufungen auf, die in der deutschen Sprache in diesem Zusammenhang bisher nicht geläufig waren.

Bereits 2021 publizierte Michel in seinem Beitrag zur neuen WHO-Klassifikation von Hörverlusten eine Übersetzung ins Deutsche [2]. Er erläuterte die Änderungen gegenüber der zuvor bestehenden WHO-Klassifikation und wies darauf hin, dass die Einstufungen anhand des Tonaudiogramms nur für epidemiologische Zwecke bei Erwachsenen verwendet werden sollen. Für die Bewertung einer Hörbeeinträchtigung und die Indikation von technischen Hörsystemen wird berechtigterweise in Deutschland hauptsächlich das Sprachaudiogramm herangezogen, wobei aber auch die WHO auf die notwendige Beurteilung von Kommunikationsschwierigkeiten bei Hintergrundgeräuschen verweist. In Deutschland gehört außerdem zum medizinisch notwendigen Ausgleich einer Behinderung, dass das binaurale Hören an den Normalzustand anzunähern ist. Dies gilt auch bei Versorgung der einseitigen Hörstörung. Das beidseitige Hören wird nach ständiger Rechtsprechung zu den Grundbedürfnissen des Menschen gerechnet [3].

## Übertragung der WHO-Klassen ins Deutsche

Eine Diskussion zur Übersetzung der englischen WHO-Klassen *normal hearing, mild hearing loss, moderate hearing loss, moderately severe hearing loss, severe hearing loss, profound hearing loss* und *complete or total hearing loss/deafness* in deutsche Begriffe wie in der Publikation von Michel [2] im Fachausschuss „Audiometrie und Qualitätssicherung“ der Deutschen Gesellschaft für Audiologie führte zur Bildung einer Arbeitsgruppe unter Einbeziehung weiterer Expertinnen und Experten. Diese verfolgte das Ziel, bezüglich der Übertragung von Tab. 1.3 zur Klasseneinteilung von Hörverlusten auf S. 38 des WRH [1] ins Deutsche einen tragfähigen Konsens zu erreichen. Bestehende, nicht auf den WHO-Kriterien beruhende Klassifikationssysteme, wie zum Beispiel die Begutachtungsrichtlinien der Lärmschwerhörigkeit, bleiben davon unberührt.

Für die Begutachtung einer Schwerhörigkeit wurden im deutschen Sprachraum bereits in den 1950er-Jahren die Oberbegriffe *Normalhörigkeit, geringgradige Schwerhörigkeit, mittelgradige Schwerhörigkeit, hochgradige Schwerhörigkeit, an Taubheit grenzende Schwerhörigkeit* und *Taubheit* eingeführt [4, 5], die u. a. in der Versorgungs-Medizin-Verordnung (VersMedV, [6]) in Beziehung zum prozentualen Hörverlust gesetzt werden. Diese grobe Einteilung kann nach Feldmann und Brusis [7] feiner abgestuft werden. Eine Gegenüberstellung der WHO-Klassen zu den deutschen Schwerhörigkeitsgraden ergab deutliche Unterschiede. So werden z. B. Patienten mit einem *severe hearing loss* nach WHO häufig einer *an Taubheit grenzenden Schwerhörigkeit* und mit einem *profound hearing loss* einer *Taubheit mit Hörresten* zugeordnet.

Aufgrund der Diskrepanzen zwischen den Klassifizierungen erschien es geboten, für die Übertragung der neuen WHO-Klassen ins Deutsche nicht die bestehenden Begriffe der Schwerhörigkeitsgrade im Deutschen zu verwenden und vor allem *profound hearing loss* nicht wie in [2] mit *hochgradiger Hörverlust* zu übersetzen.

Bei der Suche nach alternativen Begriffen zur Bezeichnung der deutschen Klasseneinteilung wurde angestrebt, ein konsistentes, auch alltagssprachlich leicht verständliches Begriffssystem für die Steigerung der Hörverluste zu wählen. Nach eingehender Diskussion wurde für die Begriffe *Normalhörigkeit, leichter Hörverlust, mäßiger Hörverlust, mäßig schwerer Hörverlust, schwerer Hörverlust, sehr schwerer Hörverlust* und *vollständiger Hörverlust/Taubheit* ein Konsens erreicht. Diese Begriffe entsprechen denjenigen von Michel [2] unter Verwendung von *mäßig schwer* für *moderately severe* und *sehr schwer* für *profound*. Im Folgenden wird der Wortlaut der im Konsens aller Koautoren erarbeiteten Übertragung ins Deutsche von Abschn. 1.3.4 des WRH wiedergegeben.

### 1.3.4 Klasseneinteilung des Hörverlusts

Um die Art und Weise zu standardisieren, in der der Schweregrad des Hörverlusts angegeben wird, hat die WHO ein auf audiometrischen Messungen basierendes Klassifizierungssystem eingeführt. Dieses System ist eine Überarbeitung eines früheren Ansatzes der WHO und unterscheidet sich von dem früheren System dadurch, dass die Bemessung der Untergrenze eines leichten Hörverlusts von 26 dB HL auf 20 dB HL herabgesetzt wurde; der Hörverlust wird in leicht, mäßig, mäßig schwer, schwer, sehr schwer oder vollständig eingeteilt; außerdem wurde ein einseitiger Hörverlust hinzugefügt. Zusätzlich zu den Klassifizierungen bietet das überarbeitete System eine Beschreibung der funktionellen Konsequenzen für die Kommunikation, die mit jedem Schweregrad einhergehen können [8]. Dieses überarbeitete Klassifizierungssystem ist in Tab. 1 dargestellt.

**Tab. 1:** Klassifikation des Hörverlusts und damit verbundene Hörerfahrungen^a^ (entspricht Tab. 1.3 des WRH)

Hörverlustklasse	Hörschwelle (HL)^b^ des besser hörenden Ohrs in Dezibel (dB)	Hörerfahrung in ruhiger Umgebung für die meisten Erwachsenen	Hörerfahrung in geräuschvoller Umgebung für die meisten Erwachsenen
*Normalhörigkeit*	< 20 dB	Kein Problem, akustische Signale zu hören	Kein oder minimales Problem, akustische Signale zu hören
*Leichter Hörverlust*	20 bis < 35 dB	Hat keine Probleme, Sprache bei Umgangssprach-Lautstärke zu verstehen	Kann Schwierigkeiten haben, Sprache bei Umgangssprach-Lautstärke zu verstehen
*Mäßiger Hörverlust*	35 bis < 50 dB	Kann Schwierigkeiten haben, Sprache bei Umgangssprach-Lautstärke zu verstehen	Hat Schwierigkeiten beim Hören und bei der Teilnahme an Gesprächen
*Mäßig schwerer Hörverlust*	50 bis < 65 dB	Hat Schwierigkeiten beim Verstehen von Sprache bei Umgangssprach-Lautstärke; keine Schwierigkeiten, lautere Sprache zu verstehen	Hat meistens Schwierigkeiten beim Verstehen von Sprache und bei der Teilnahme an Gesprächen
*Schwerer Hörverlust*	65 bis < 80 dB	Versteht Sprache bei Umgangssprach-Lautstärke meistens nicht; kann Schwierigkeiten haben, lautere Sprache zu verstehen	Hat extreme Schwierigkeiten, Sprache zu verstehen und an Gesprächen teilzunehmen
*Sehr schwerer Hörverlust*	80 bis < 95 dB	Hat extreme Schwierigkeiten, lautere Sprache zu verstehen	Kann Sprache bei Umgangssprach-Lautstärke nicht verstehen
*Vollständiger Hörverlust/Taubheit*	95 dB oder höher	Kann Sprache und die meisten Umweltgeräusche nicht hören	Kann Sprache und die meisten Umweltgeräusche nicht hören
*Einseitiger Hörverlust*	< 20 dB auf dem besser hörenden Ohr, 35 dB oder höher auf dem schlechteren Ohr	Hat möglicherweise kein Problem, außer bei Beschallung von der schlechter hörenden Seite. Möglicherweise Schwierigkeiten bei der Lokalisierung von Schallquellen	Hat möglicherweise Schwierigkeiten beim Sprachverstehen und bei der Teilnahme an Gesprächen sowie bei der Lokalisation von Schallquellen

*WRH *World Report on Hearing

^a^ Die Klassifizierung und Einstufungen sind für epidemiologische Zwecke gedacht und gelten für Erwachsene. Bei der Anwendung dieser Klassifizierung sind die folgenden Punkte zu beachten:

– Die audiometrischen Deskriptoren (z. B. Kategorie, Mittelwert der Tonhörschwellen) bieten zwar eine nützliche Zusammenfassung der Hörschwellen einer Person, sollten aber nicht als alleinige Determinante für die Bewertung der Behinderung oder die Bereitstellung von Interventionen, einschließlich Hörgeräten oder Cochlea-Implantaten, verwendet werden.

– Die Fähigkeit, Sinustöne mit Kopfhörern in einer ruhigen Umgebung wahrzunehmen, ist an sich kein zuverlässiger Indikator für eine Hörbehinderung. Audiometrische Deskriptoren allein sollten nicht als Maßstab für Kommunikationsschwierigkeiten bei Hintergrundgeräuschen herangezogen werden, die das Hauptproblem von Menschen mit Hörverlust darstellen.

– Ein einseitiger Hörverlust kann bei jedem Grad der Asymmetrie eine große Herausforderung für den Betroffenen darstellen. Er erfordert daher eine angemessene Aufmerksamkeit und Intervention auf der Grundlage der von der Person erlebten Schwierigkeiten.

^b^ „Hörschwelle“ ist der Mittelwert der Mindestpegel, die auf dem besser hörenden Ohr bei den Frequenzen 500, 1000, 2000 und 4000 Hz wahrgenommen werden können [8–10]

Die in Tab. 1.3 des WRH verwendeten Klassifikationen folgen den Empfehlungen der International Classification of Functioning, Disability and Health (ICF; Infobox), die von der WHO im Jahr 2001 vorgeschlagen wurde. Gemäß der ICF hat eine Person mit der geringsten Beeinträchtigung des Hörvermögens eine potenzielle „Behinderung“. Die ICF definiert den Gesundheitszustand einer Person anhand von drei Dimensionen, die in der Infobox dargestellt sind [11]. Nach der ICF wird die Behinderung nicht nur durch den Hörverlust der Person bestimmt, sondern auch durch das physische, soziale und einstellungsbedingte Umfeld, in dem die Person lebt, und die Möglichkeit des Zugangs zu hochwertiger Ohr- und Hörgeräteversorgung. Eine schwerhörige Person, die keinen Zugang zu einer Hörversorgung hat, muss daher mit weitaus größeren Einschränkungen im täglichen Leben und somit mit einem höheren Grad an Behinderung rechnen.

#### Infobox Internationale Klassifikation der Funktionsfähigkeit, Behinderung und Gesundheit [11]

Die International Classification of Functioning, Disability and Health (ICF) ist der WHO-Rahmen für die Messung von Gesundheit und Behinderung sowohl auf individueller als auch auf Bevölkerungsebene. Die ICF definiert den Gesundheitszustand einer Person anhand von drei Dimensionen:

(i) *Beeinträchtigung*: Sie bezieht sich auf die Funktion oder Form des Körpers (im Fall des Hörvermögens als „Hörverlust“ bezeichnet).

(ii) *Aktivitätseinschränkung*: Sie bezieht sich auf die persönliche Funktionsebene (früher als „Behinderung“ bezeichnet).

(iii) *Teilhabeeinschränkung*: Sie bezieht sich auf die psychosoziale Funktion (in früheren Versionen der ICF als „Handicap“ bezeichnet).

Der Begriff „Behinderung“ umfasst alle Probleme oder Schwierigkeiten, auf die eine Person mit Hörverlust bei der Durchführung alltäglicher Aktivitäten oder Situationen stößt, wie z. B. bei der Selbstversorgung, beim Schulbesuch oder bei der Arbeit. Der Begriff „Behinderung“ im Zusammenhang mit Hörverlust bezieht sich auf die erfahrenen Beeinträchtigungen, Beschränkungen und Einschränkungen (körperlich, sozial oder einstellungsbedingt). Da Funktionsfähigkeit und Behinderung durch den Kontext beeinflusst werden, enthält die ICF auch eine Liste von Umweltfaktoren, die zu den Schwierigkeiten von Menschen mit Hörverlust beitragen.

## Fazit für die Praxis

- Die WHO-Klassen zum Hörverlust dienen vorrangig epidemiologischen Zwecken und ermöglichen die internationale Vergleichbarkeit audiologischer Messungen in Studien.
- Die WHO-Klassenbezeichnungen werden im Konsens der Arbeitsgruppe mit *Normalhörigkeit, leichter Hörverlust, mäßiger Hörverlust, mäßig schwerer Hörverlust, schwerer Hörverlust, sehr schwerer Hörverlust* und *vollständiger Hörverlust/Taubheit* übersetzt.
